# Fatal pulmonary sporotrichosis caused by *Sporothrix brasiliensis* in Northeast Brazil

**DOI:** 10.1371/journal.pntd.0008141

**Published:** 2020-05-26

**Authors:** Manoella do Monte Alves, Eveline Pipolo Milan, Walicyranison Plinio da Silva-Rocha, Alexandre Soares de Sena da Costa, Bruno Araújo Maciel, Pedro Henrique Cavalcante Vale, Paulo Roberto de Albuquerque, Soraia Lopes Lima, Analy Salles de Azevedo Melo, Anderson Messias Rodrigues, Guilherme Maranhão Chaves

**Affiliations:** 1 Departamento de Infectologia, Instituto de Medicina Tropical, Universidade Federal do Rio Grande do Norte, Natal, Rio Grande do Norte, Brasil; 2 Laboratório de Micologia Médica e Molecular, Departamento de Análises Clínicas e Toxicológicas, Universidade Federal do Rio Grande do Norte, Natal, Rio Grande do Norte, Brasil; 3 Hospital Universitário Onofre Lopes, Universidade Federal do Rio Grande do Norte, Natal, Rio Grande do Norte, Brasil; 4 Laboratório Especial de Micologia, Disciplina de Infectologia,Universidade Federal de São Paulo, São Paulo, Brasil; 5 Laboratório de Fungos Patogênicos Emergentes, Divisão de Biologia Celular, Departamento de Microbiologia, Imunobiologia e Parasitologia, Escola Paulista de Medicina, Universidade Federal de São Paulo, São Paulo, Brasil; Faculty of Science, Ain Shams University (ASU), EGYPT

## Abstract

**Background:**

A relevant case of pulmonary sporotrichosis due to *Sporothrix brasiliensis* is reported in a 50-year-old immunocompetent woman who had no history of skin trauma, but was in close contact with several stray cats at her nap time. The patient was hospitalized after 7 months of illness. The survey was conducted for pulmonary tuberculosis, an endemic disease in Brazil. She presented multiple central pulmonary nodules images, with central cavitation.

**Methodology/Principal findings:**

The patient bronchoalveolar lavage was cultured and *Sporothrix* sp. growth was obtained. Then, the isolate (LMMM1097) was accurately identified to the species level by using species-specific polymerase chain reaction (PCR). Molecular diagnosis revealed that the emerging species *Sporothrix brasiliensis* was the agent of primary pulmonary sporotrichosis and the patient was treated with Amphotericin B lipid complex, but presented severe clinical symptoms and the fatal outcome was observed at day 25 after hospitalization.

**Conclusions/Significance:**

Our report adds important contributions to the clinical-epidemiological features of sporotrichosis, showing the geographic expansion of the agent within different regions of Brazil and a rare clinical manifestation (primary pulmonary sporotrichosis) caused by the emerging agent *S*. *brasiliensis* in an immunocompetent female patient.

## Author summary

Sporotrichosis is a fungal infection caused by different species belonging to the genus *Sporothrix*. In the past, the disease was usually associated with armadillo hunting and horticulturists, acquired after traumatic inoculation. Nevertheless, from 1997 to 2011, 4,188 human cases of sporotrichosis associated with feline transmissions were recorded in Rio de Janeiro, Brazil. Since then, the disease became an epidemic that is currently spreading within different regions of the country. Nevertheless, pulmonary sporotrichosis, specifically after conidial inhalation (without traumatic inoculation) is still a rare entity. In the present study, a female patient had the habit to have a nap at her workplace in a small room full of stray cats, daily. Tuberculosis was investigated, but the laboratory tests were negative. Then, pulmonary aspergillosis was suspected and Amphotericin B was initiated. A culture of the bronchoalveolar lavage was performed, which evidenced *Sporothrix* sp. growth. The final species identification was *Sporothrix brasilensis*, as demonstrated by PCR. However, the patient evolved to death. This finding reinforces the idea of exploring different possibilities of etiological agents involved in pulmonary infections, specific in areas of the globe where zoonotic sporotrichosis transmitted by cats have been reported.

## Introduction

Sporotrichosis is a granulomatous fungal infection that affects humans and animals and presents a subacute or chronic character. The disease is classically associated with traumatic inoculation of *Sporothrix* spp. propagule into the host tissue [1]. Infection usually occurs after traumatic inoculation with plant debris, soil and contaminated organic material (sapronotic route). Zoonotic transmission has been recently described following bites or scratches, mainly by cats [2]. Lesions are usually restricted to the skin, subcutaneous cellular tissue, and adjacent lymphatic vessels. However, under some circumstances, this fungus can disseminate to other organs (extracutaneous form), and on rare occasions, inhalation of conidia may lead to systemic disease [3].

Species frequently involved in human’s diseases cluster together in a pathogenic clade including *S*. *brasiliensis*, *S*. *schenckii*, *S*. *globosa* and *S*. *luriei*, clearly separated from the *Sporothrix*/*Ophiostoma* species complex that belongs to the environmental clade (rarely associated with human and animals’ disease). Less frequent agents present mid-to-low pathogenic potential to mammals and are assembled in the *S*. *pallida* complex, including *S*. *chilensis*, *S*. *mexicana* and *S*. *pallida* [4].

*S*. *brasiliensis* has recently been described as more virulent than *S*. *sckenckii sensu stricto* in a murine model of subcutaneous infection. The mice infected with *S*. *brasiliensis* developed more extensive and long-lasting local lesion and maintained a higher liver and spleen burden throughout the experiment, in addition to higher cytokine levels, whereas *S*. *schenckii*-infected mice developed small granulomas with lower fungal burden and less extensive inflammatory cells [5].

After traumatic inoculation, sporotrichosis may be presented as a polymorphic disease, ranging from localized to disseminated manifestations. The classical clinical forms involve lymphocutaneous and fixed cutaneous sporotrichosis which account by the vast majority of the cases [1, 6–9]. However, the number of atypical clinical presentations including pulmonary, osteoarticular, meningeal and disseminated sporotrichosis has increased, mainly affecting immunocompromised patients with HIV infection, diabetes, alcoholism and other immunosuppression forms [9–10]. Primary pulmonary sporotrichosis is a rare presentation which occurs after inhaling fungal conidia present in the environment, with subsequent inoculation into structurally abnormal lungs [8, 11].

In the last decade, the increased incidence of sporotrichosis cases in tropical and subtropical areas has been reported [12–13]. The incidence of sporotrichosis varies among Latin American countries, with endemic areas in Brazil, Colombia, Costa Rica, Guatemala, Mexico and Uruguay, being very rare in Chile. Its prevalence ranges from 0.1 to 0.5% in Brazil, Colombia, El Salvador, Mexico, Uruguay and Venezuela; and 0.01 to 0.02% in Argentina, Ecuador and Panama [14–16]. Nevertheless, as sporotrichosis is not a notifiable disease, these data may not represent the reality. A recent review reported 5,814 cases of sporotrichosis in Brazil, estimating that 88% of them were due to *S*. *brasiliensis*, 9% to *S*. *schenckii*, and the others to unusual species (*S*. *globosa* and *S*. *mexicana*) [17]. Another study reported the diagnosis of sporotrichosis for 14 Brazilian states. However, the massive number of cases occurred within the South and Southeast regions [18].

The purpose of this article is to report a case of primary pulmonary sporotrichosis without cutaneous manifestations, lately diagnosed in a likely healthy immunocompetent female patient from the Northeast region of Brazil. The patient presented fatal clinical evolution, despite antifungal treatment with Amphotericin B lipid complex. To the best of our knowledge, this is the first fatal case of primary pulmonary sporotrichosis due to *S*. *brasiliensis* in the literature.

### Case report

**Fig 1:**
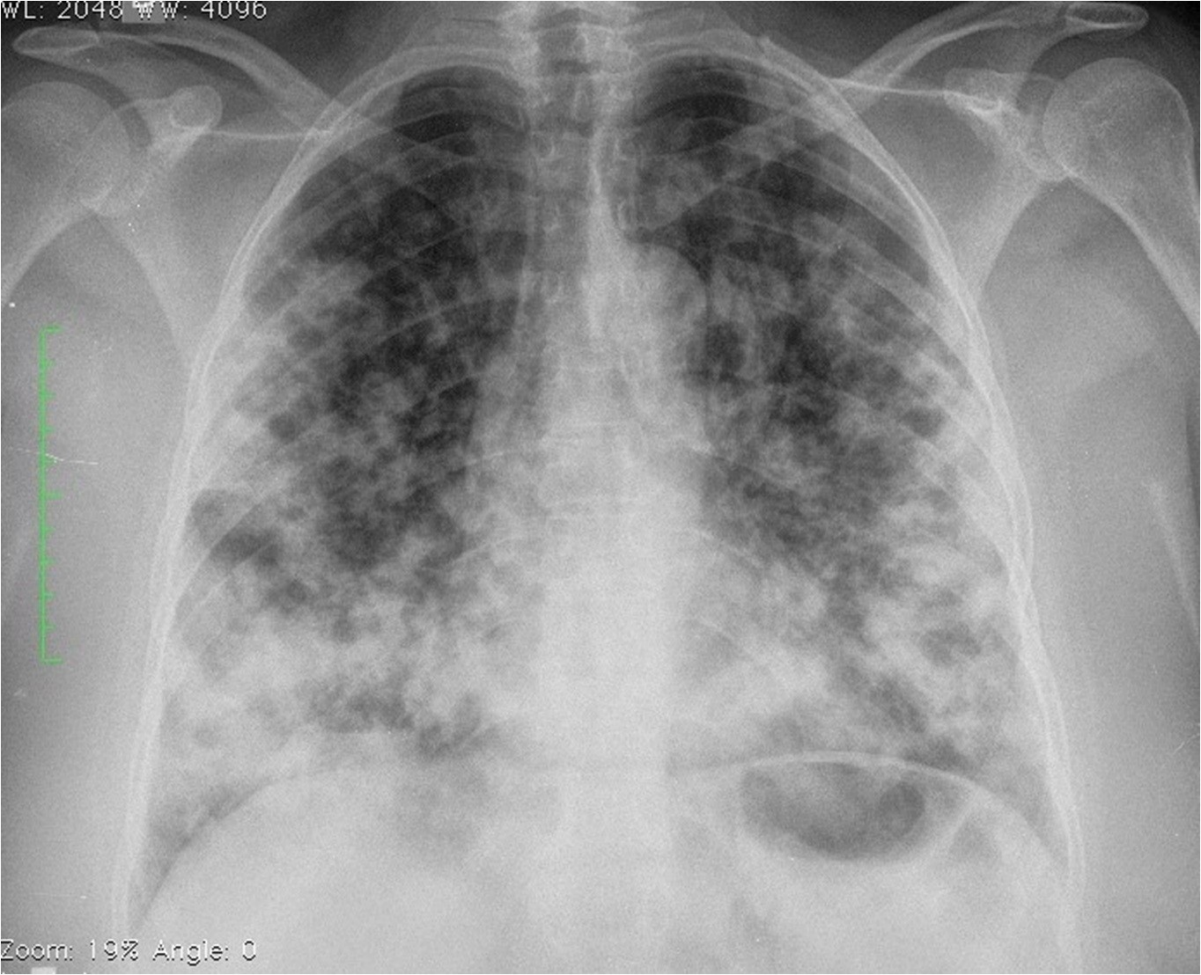
Chest X-ray of a patient with primary pulmonary sporotrichosis showing multiple bilateral pulmonary nodules.

**Fig 2:**
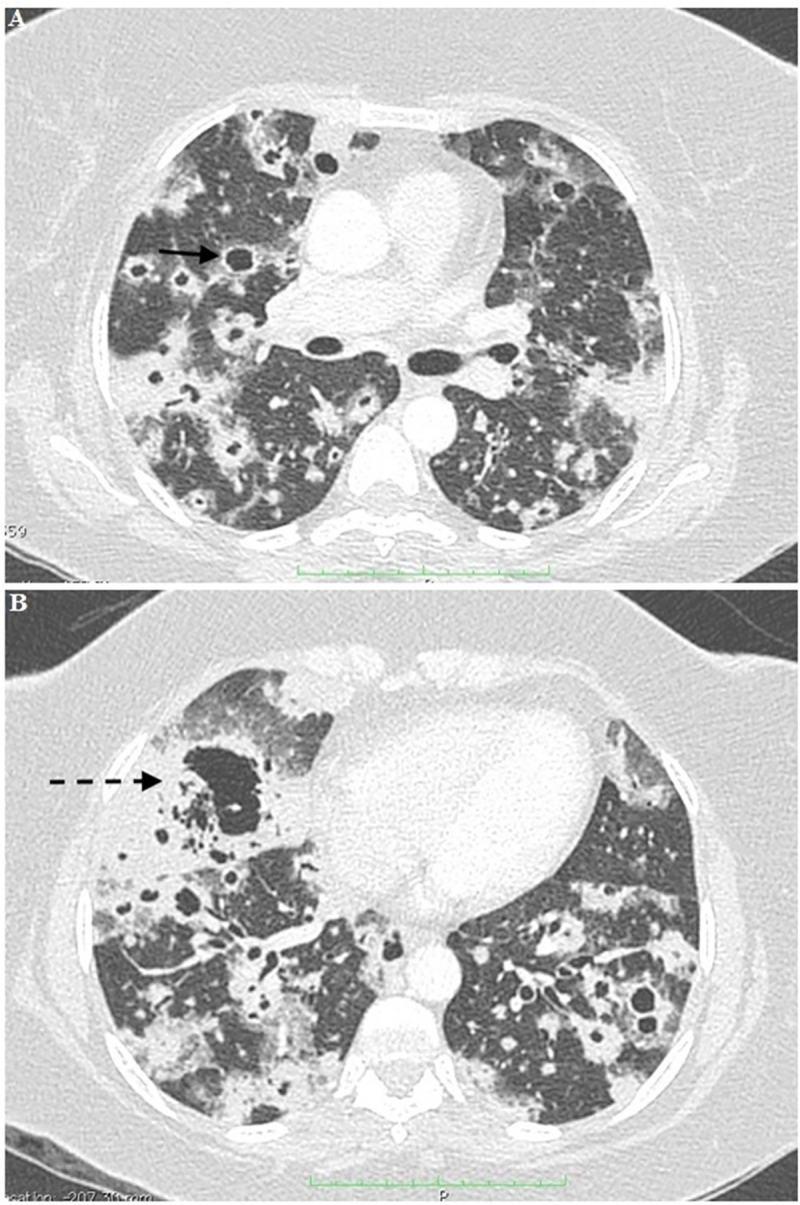
Chest computed tomography (CT) of a patient showing: A) Multiple nodular images with central necrosis and thickened walls, surrounded by a ground-glass halo dispersed by the parenchyma (black arrow). B) Confluent nodulations with a large mass with apparent central cavitation in the right lobe (dashed black arrow).

A 50-years-old non-smoker and immunocompetent female from Natal, Rio Grande do Norte state, Northeast Brazil was admitted to our tertiary University hospital on the 6^th^ of December 2016 with a 7-month history of snoring, cough with hyaline expectoration, progressive dyspnoea after moderate exertion and weight loss of 5 kg. She was empirically and clinically treated with levofloxacin 500 mg/day and Fluconazole 150 mg/day, both for 10 days, without improvement. The patient reported she used to live with her husband and daughter in a concrete masonry construction of an urban area and had an apparently healthy dog. During a period of 15 years, she was exposed to filamentous fungi and dust at the retail shop where she worked and used to rest after lunch in a small enclosed room with several stray cats she used to feed. The patient had no morbid antecedents. At the clinical examination, she presented good general condition, breathing normally, without visible respiratory exertion. At the respiratory evaluation, she presented bilateral vesicular murmurs, Velcro-type rales and bilateral diffuse wheezing. No abnormalities were found at cardiac auscultation, abdominal examination and skin. A complete blood count showed white blood cells of 8650/mm^3^ with 71% neutrophils and 21% lymphocytes. Her haemoglobin was 15.50 g/dl, mean corpuscular volume 79.8 fl, and platelets 257,000/mm^3^. High sensitivity C-reactive protein was 120 mg/L and lactate dehydrogenase was 366 U/L. Chest X-ray revealed multiple bilateral pulmonary nodules (Fig 1). Research for acid-fast bacilli and rapid molecular test for *Mycobacterium tuberculosis* (GenXpert) were negative but she had a positive sputum culture for *Aspergillus* sp. A chest computed tomography (CT) revealed multiple nodular images, bilaterally, some with central necrosis and thickened walls (Fig 2A), surrounded by a ground-glass halo dispersed by the parenchyma, of varying sizes (0.3 cm to 2.8 cm). In the right lobe, nodulations are confluent and form a large mass with apparent central cavitation (Fig 2B), suggesting fungal infection. Bronchoscopy was performed and antifungal therapy with Amphotericin B lipid complex 5 mg/kg/day was started on the 10th day of hospital admission (DHA), considering chronic pulmonary aspergillosis (12/16/16). Due to worsening cough, respiratory rate increase, and maintenance of wheezing, prednisone 0.5 mg/kg/day was started at the same day. Serologies for hepatitis B and C, HIV and syphilis were non-reactive. Urinary protein immunoelectrophoresis were normal. IgA, IgG and IgM serum dosages were also normal. Normal mammography and CT scan of the abdomen and pelvis for screening of primary neoplasms presented no abnormalities. Facial sinus CT and P-ANCA/C-ANCA dosing for the basal granulomatous disease were considered negative. Our patient presented significant hypoxemia after 5 days of Amphotericin B lipid complex administration, requiring support from the intensive care unit (ICU), at the 14^th^ DHA (12/20/16). It was hypothesized the possibility of hospital-acquired bacterial pneumonia or pulmonary thromboembolism and piperacillin-tazobactam 4.5g every 6 hours plus low molecular weight heparin 1mg/day every 12 hours were started. Prednisone was substituted by intravenous methylprednisolone. CT angiography was negative for pulmonary thromboembolism but showed disease progression and multiple consolidation areas. She evolved to acute respiratory failure requiring orotracheal intubation and mechanical ventilation at the 16th DHA (12/22/16). The day after, the patient developed acute renal insufficiency and subsequently septic shock, and noradrenalin and haemodialysis were started. A mycological examination was performed and at the direct examination (KOH 20%) of the bronchial lavage, rare single-budding yeast cells were visualized. The culture evidenced growth of *Sporothrix* sp. after 5 days of incubation. The smear collected from the bronchoalveolar lavage fluid resulted in a material consisting of a moderate number of macrophages among a small number of lymphocytes, polymorphonuclear, red blood cells and ciliary columnar cells. Cells with bulky nuclei and vacuolated cytoplasm were occasionally observed. Dosage of galactomannan was negative. The patient continued to develop severe signs of sepsis, with septic shock and need for vasoactive drugs at 17th DHA (12/23/16). At 18th DHA (12/24/16) she presented signs of acute renal injury and Amphotericin B was switched to itraconazole 200 mg every 8 hours. At 20th DHA hydrocortisone 300 mg/day was started due to a refractory shock. The patient died at 25th DHA, on the day 15 after starting antifungal therapy (12/31/2016; Table 1).

**Table 1:** Timeline of a female patient clinical evolution diagnosed with primary pulmonary sporotrichosis in Natal city, Rio Grande do Norte State, Northeast Brazil.

Period of time	Week 1	Week 2	Week 3	Week 4
**Empirical antibacterial (LEV) and antifungal (FLU) therapy**	X	X		
**Multiple bilateral pulmonary nodules (Chest X-ray)**	X			
***Aspergillus* sp. positive culture on sputum**	X			
**Pulmonary nodular images and central necrosis (CT)**	X			
**Antifungal therapy (AmB)**		X		
**Corticosteroid treatment (PRS)**		X		
**Antibacterial therapy (PTAZ)**		X		
**ICU admission**		X		
**Intravenous corticosteroid**		X		
**Acute respiratory failure (mechanical ventilation)**		X		
**Acute renal insufficiency and subsequently septic shock**			X	
**Hemodialysis**			X	
***Sporothrix brasiliensis* BAL positive direct examination and culture**			X	
**AmB to ICZ replacement**			X	
**Death**				X

^LEV (Levofloxacin); FLU (Fluconazole); CT (Computed Tomography); AmB (Amphotericin B); PRS (Prednisone);^

^PTAZ (Piperacilin + tazobactan); ICU (Intensive care Unit); ICZ (Itraconazole); BAL (Bronchoalveolar lavage)^

## Methods

### Ethics statement

All clinical and demographic data of the patient were collected in accordance with the Local Research Ethics committee from the “Liga Norte Riograndense Contra o Câncer Hospital”, approved under number 042/042/2012.Written informed consent was obtained from the patient´s daughter for publication of this case report and any accompanying images.

### Direct examination, culture and microculture

The bronchoalveolar lavage sample was centrifuged at 5000 RPM, 25°C during 10 minutes. Mycological direct examination of the clinical sample was performed by clarification with potassium hydroxide (KOH 20%) for 30 minutes. The sample was analyzed with optical microscopy (400x of magnification). Subsequently, 20 μL aliquots of the precipitate were inoculated at seven equidistant spots on the surface of Mycosel Agar (BD, NJ, USA). The Petri dish was incubated at 25°C and observed daily up to a month. Transition to the yeast phase was performed with further colony subculture at 37°C for another 5–10 days on the same culture medium.

The macromorphological characteristics of the colonies, such as diameter, surface aspect and melanin production were analyzed. The micromorphological aspects including septation, hyphal presence or absence of pigment and type of conidiogenesis were observed for the filamentous phase. The presence of budding cigar-shaped yeast cells was observed for yeast colonies.

### Molecular identification

The yeast phase of the isolate obtained (namely LMMM1097) was cultured on Mycosel agar during seven days at 37°C and DNA was extracted using The PrepMan Ultra Sample Preparation Reagent (Applied Biosystems), according to the manufacturer's instructions. DNA concentration and purity were determined with a NanoDrop spectrophotometer (Thermo Fisher Scientific). DNA was used for polymerase chain reaction (PCR) with species-specific primers that target the calmodulin gene (*CAL*) as previously described [19]. Briefly, reactions were performed in 25 μL of final volume, including 12.5 μL PCR Master Mix (Promega Corporation), consisting of 3 mM MgCl_2_, 400 mM each dNTPs, and 50 U/mL Taq Polymerase; 9.5 μL water, 1 μL each of forward and reverse primers (10 pmol/μL; Integrated DNA Technologies, USA), and 1 μL of target DNA [100 ng/μL]. The following primers were used: Sbra-F [5´- CCC CCG TTT GAC GCT TGG- 3´], Sbra-R [5´CCC GGA TAA CCG TGT GTC ATA AT -3´], Ssch-F [5´- TTT CGA ATG CGT TCG GCT GG—3´], Ssch-R [5´- CTC CAG ATC ACC GTG TCA—3´], Sglo-F [5´- CGC CTA GGC CAG ATC ACC ACT AAG—3´], Sglo-R [5´- CCA ATG TCT ACC CGT GCT- 3´], Smex-F [5´-TCT CTG CCG ACA ATT CTT TCT C—3´], and Smex-R [5´- GGA AAG CGG TGG CTA GAT GC—3´] [19]. PCR products were size-separated by agarose gel electrophoresis and the gel was stained in a 0.5 μg/mL ethidium bromide buffer solution (TAE).

## Results

### Direct examination, culture and microculture

**Fig 3:**
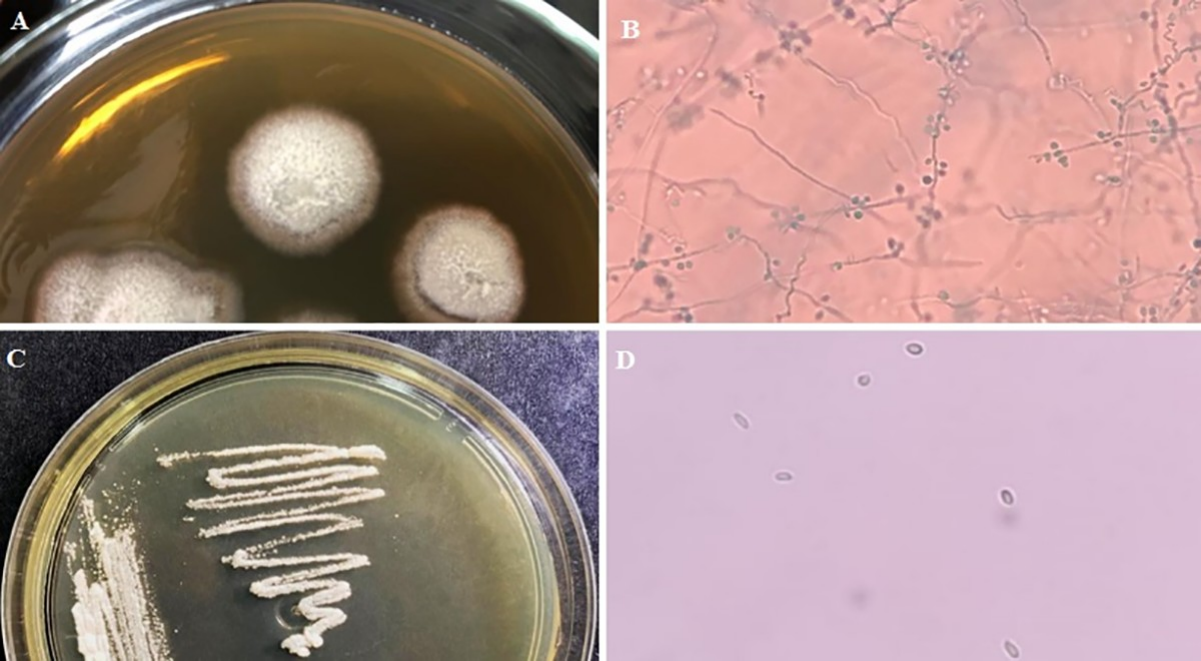
*Sporothrix brasilensis* strain LMMM1097 mycological culture on Mycosel Agar. A) Macromorphological aspect of filamentous colonies after 5 days of incubation at 28°C. B) Micromorphological aspect of filamentous colony fragment observed with optical microscopy after lactophenol cotton blue staining C) Macromorphological aspect of yeast colonies after 5 days incubation at 37°C. D) Micromorphological aspect of yeast colony fragment observed with optical microscopy mounted with saline solution.

Direct examination with KOH was performed with optical microscopy. It was observed the presence of epithelial cells, leukocytes and rare budding yeasts. After five days of incubation, a filamentous fungi colony was observed at 28°C incubation and a yeast-like colony was observed after 37°C incubation for conversion to the yeast phase. Colonies at the filamentous form (28°C) were wrinkled, dirty whitish, reverse grey to brownish-black. Microscopically, hyaline and septate hyphae and conidia in a bouquet arrangement were found. Creamy and pale-skinned colonies and multilaterally budding cigar-shaped yeast cells form were observed at 37°C. The fungal colony obtained in culture was identified as *Sporothrix* sp. (Fig 3A to 3D).

### Molecular identification

The strain LMMM1097 isolated from the bronchoalveolar lavage sample was subjected to molecular identification. After PCR reaction and subsequent electrophoresis, a 469 bp DNA fragment product was observed using primers Sbra-F and Sbra-R, leading to the identification of *S*. *brasiliensis*, according to Rodrigues *et al*. [19].

## Discussion

Primary pulmonary sporotrichosis is a rare entity, transmitted through environmental conidia inhalation [7]. Pulmonary sporotrichosis may be a disseminated infection secondary to cutaneous sporotrichosis acquired by traumatic inoculation [11]. Secondary pulmonary sporotrichosis (multifocal sporotrichosis) may present multiple organs involvement, specifically in immunocompromised patients [6, 20].

Aung et al. [20] performed a systematic review of pulmonary sporotrichosis cases published in English from 1960 to 2010. The authors identified 86 cases, where 64 of them (74.4%) were primary pulmonary sporotrichosis and 22 (25.6%) multifocal disease. Of the 86 patients, 72 (83.7%) were males and the mean age was close to 50 years. *S*. *schenckii sensu stricto* were isolated from 83 patients, whereas none of them were identified as *S*. *brasiliensis*. Only two out of 86 cases were reported in Brazil, where *S*. *brasiliensis* is endemic and highly frequent in humans and cats [4, 12, 21–22]. In fact, the proposal of the novel molecular species *S*. *brasiliensis* occurred only in 2007 [23], and we cannot rule out the possibility that any (or both) of the two Brazilian reported cases was caused by this species.

The epicenter of the Brazilian epidemic zoonotic sporotrichosis is Rio de Janeiro. Orofino-Costa *et al*. [24] reported a case of pulmonary sporotrichosis due to *S*. *brasiliensis* in an HIV negative male living in Rio de Janeiro city who developed multiple pulmonary cavitations and skin abscesses mimicking tuberculosis. In this particular report, the patient responded well to Amphotericin B, followed by itraconazole, except the skin lesions that had to be surgically drained to obtain cure [24]. From an epidemiological point of view, *S*. *brasiliensis* is the main agent during epizooties in cats leading to zoonotic transmission to humans. We report a case of primary pulmonary sporotrichosis due to *S*. *brasiliensis* in an area spanning a nearly 2,000 km of distance from the epicenter in Rio de Janeiro, showing the emerging characteristic of this species in other parts of Brazil. The number of atypical cases due to *S*. *brasiliensis* has been increasing [25] and its high virulence to mammals has been constantly reported [26–27], including more aggressive manifestations in humans [28]. On the other hand, the species included in the environmental clade, such as *S*. *chilensis*, *S*. *mexicana* and *S*. *pallida* [29–30], as well as to *S*. *globosa*, (clinical clade) are attenuated in virulence [31].

Aung et al. [20] reported the following risk factors for primary pulmonary sporotrichosis: male sex, middle age, smoking, chronic obstructive pulmonary disease, chronic use of corticosteroids, immunosuppressive disease and alcoholism [8, 10, 20]. Primary pulmonary form presents clinically similar to tuberculosis, with fatigue, weight loss, cough, low fever, mediastinal lymphadenitis, cavitation, fibrosis and rarely massive hemoptysis. Apical lesions occur in 85% of cases [7, 11, 32], whereas in multifocal sporotrichosis, pulmonary involvement is secondary to spread of infection, acquired after traumatic injury where bibasal or diffuse reticulonodular infiltrate are more frequently found. It presents with non-cavitary lesions and usually occurs in immunosuppressed patients [32].

The clinical condition suggested invasive pulmonary aspergillosis and hypothesis was strengthened due to the occurrence of *Aspergillus* sp. positive sputum culture. However, it is well known that it is not possible to differentiate among colonization and infection when *Aspergillus* spp. are isolated from respiratory samples [33]. Invasive pulmonary aspergillosis was also excluded because the dosage of galactomannan was negative.

It is important to consider that in October 2016, in the city of Natal, Rio Grande do Norte state, the first feline sporotrichosis case was diagnosed and from October to December 2016, four human cases of zoonotic sporotrichosis were diagnosed by our group: 03 of them were lymphocutaneous form, while the last patient had cutaneous-disseminated form, all secondary to trauma caused by cats also diagnosed with sporotrichosis. Our patient did not report any trauma or contact with soil or plant debris. However, she used to work for 15 years in a handicraft center where several stray cats were usually present. In addition, her lunch and resting room was full of felines which used to stay there for a long time, because other workers usually used to feed those animals.

The patient only potential exposure was probably conidia inhalation at her work environment. We consider, therefore, that our patient developed primary pulmonary sporotrichosis without skin disease. Her only typical risk factor for this form of the disease was the age. The only comorbidity seen in the patient was overweight. The fact that her CT scan revealed multiple nodular images, bilaterally, with central necrosis (unusual for primary pulmonary sporotrichosis) and thickened walls and a large mass with apparent central cavitation in the right lobe may have been due the fact *S*. *brasiliensis* has been considered more virulent than *S*. *schenckii sensu stricto* in murine models of infection [5]. In addition, frequent exposition to fungal propagules and high inoculum size may have influenced the establishment of infection.

Despite treatment with Amphotericin B lipid complex, the patient progressed with worsening of clinical condition, culminating with ICU admission, mechanical ventilation, hemodialysis and use of vasoactive drugs, until death in the 25th DHA. In the systematic review of Aung et al, [20], the authors observed therapeutic success in 15 of 34 (44.1%) patients treated solely with Amphotericin B. Amphotericin B lipid complex treatment was administered to the patient of the present study and, according to Kauffman et al. [34], in severe cases, lipid formulation of Amphotericin B as initial treatment should be performed, whether it is liposomal, lipid complex or colloidal dispersion. It is important to note that our patient used corticosteroids from the 10th DHA, which may have corroborated for fungal dissemination and poor response to antifungal treatment.

This case emphasizes the role of differential diagnosis of emerging diseases when there are new outbreaks, even with atypical presentations. Brazilian and bordering countries public health agencies must be aware of primary pulmonary sporotrichosis to better prevent, diagnose, manage the disease and control outbreaks. To the best of our knowledge, this is the first fatal case report of primary pulmonary sporotrichosis due to *S*. *brasiliensis* published in the literature.
